# The *TXA2R* rs1131882, *P2Y1* rs1371097 and *GPIIIa* rs2317676 three-loci interactions may increase the risk of carotid stenosis in patients with ischemic stroke

**DOI:** 10.1186/s12883-019-1271-0

**Published:** 2019-03-26

**Authors:** Xingyang Yi, Jing Lin, Qiang Zhou, Ruyue Huang, Zhenxiao Chai

**Affiliations:** 1 Department of Neurology, People’s Hospital of Deyang City, Deyang, 618000 Sichuan China; 2 Department of Neurology, the Third Affiliated Hospital of Wenzhou Medical University, No 108 Wanson road, Ruan City, Wenzhou, 325200 Zhejiang China

**Keywords:** Carotid atherosclerosis, Genetic polymorphism, Carotid stenosis, Platelet activation, GMDR

## Abstract

**Background:**

The genetic risk factors for carotid stenosis are not fully understood. The aim of this study is to investigate the relationship between variants in platelet activation-relevant genes and carotid stenosis in patients with ischemic stroke (IS).

**Methods:**

Eleven variants of platelet activation-relevant genes, aggregates of platelet-leukocyte, and platelet aggregation were examined in 236 IS patients with carotid stenosis and 378 patients without carotid stenosis. High-resolution B-mode ultrasound was used to assess carotid stenosis. Generalized multifactor dimensionality reduction (GMDR) methods were applied in analyzing gene-gene interactions to determine whether there was any interactive role of assessed variants in affecting risk of carotid stenosis.

**Results:**

Platelet aggregation and aggregates of platelet-leukocyte showed higher value in patients with carotid stenosis, compared with patients without carotid stenosis. Excluding potential disturbance variables, these 11 variants were not associated with carotid stenosis. However, according to the GMDR analysis, gene-gene interactions among *TXA2R* rs1131882, *P2Y1* rs1371097 and *GPIIIa* rs2317676 had a synergistic influence on carotid stenosis. The high-risk interactions between the three variants showed a relationship with higher platelet activation, and have independent associations with risk of carotid stenosis (OR = 2.72, 95% CI: 1.28–7.82, *P* = 0.001).

**Conclusion:**

The interactions among rs1131882, rs1371097 and rs2317676 perhaps increase the risk of symptomatic carotid stenosis, and maybe a potential marker for carotid stenosis. In this study, the combinatorial analysis made good use in elucidating complex risk factors in the heredity of carotid stenosis.

## Background

In China, stroke has become one of the common causes of disability and mortality [1]. Carotid atherosclerosis is a leading cause of stroke. Severe internal carotid artery stenosis (≥ 50%) is often accompanied by carotid atherosclerosis and emboli, thereby preventing blood flow to the brain, which increases the risk of stroke as well [2, 3]. Moreover, a well-analyzed report has proved that the severe carotid stenosis has a serious impact on stroke recurrence or cardiovascular outcomes, and its value has been fully confirmed in the stroke population [4, 5]. Therefore, it is necessary to clarify the etiology of carotid stenosis within the context of stroke for preventing stroke.

Atherosclerosis is a complex inflammatory disorder. The genetic etiology of carotid stenosis has been proposed. Variants in inflammation and endothelial functions genes have been reported to play roles in atherosclerosis pathogenesis in the Dominican population [6]. In 2016, our previous studies illustrated the variants in Cytochrome P450 genes and eicosanoid genes were independently associated with carotid stenosis [7, 8]. However, until now the genetic etiology of carotid stenosis has not been sufficiently recognized.

Platelet activation (e.g. platelet-leukocyte aggregates and platelet aggregation) has an essential role in triggering arterial thrombosis and in promoting atherosclerosis [9, 10]. Thromboxanes A2 (TXA2), platelet membranes receptors (P2Y12, P2Y1), TXA2 receptor (TXA2R) and fibrinogen receptor may play key roles in the process of platelet activation [11–13]. Arachidonic acid (AA) is metabolized to prostaglandin H by cyclooxygenase. Prostaglandin H is commonly metabolized through thromboxane synthase (TXAS) to TXA2 [14]. TXA2 is a vasoconstrictor and platelet activator, which promotes platelet aggregation and atherothrombosis [11]. Combination of TXA2 and TXA2R (which be considered as the reporter of TXA2) could increase platelet activation [15]. The fibrinogen receptor such as glycoprotein IIb and glycoprotein IIIa (namely GPIIb and GPIIIa) constitute the final pathway for platelet activation and aggregation [13]. Our previous studies confirmed that single nucleotide polymorphisms (SNPs) in these platelet activation-relevant genes were associated with the risk of ischemic stroke (IS) and responsiveness to antiplatelet medications [16–18]. However, the associations between variants of platelet activation-relevant genes with carotid stenosis have not been well understood. Furthermore, the investigation of multi-gene interactions is necessary to elucidate genetic mechanisms for complex diseases such as carotid stenosis by using the generalized multifactor dimensionality reduction (GMDR) approach [19]. However, the GMDR approach is rarely used to investigate complex genetic risk factors of carotid stenosis.

In current study, we measured 11 variants of platelet activation-relevant genes in IS patients with and without carotid stenosis, and investigated the associations of these variants and interactions among these variants with carotid stenosis in Chinese population.

## Methods

### Ethics statement

This study protocol was reviewed and approved by the Ethics Committee of the Ethics Committee of the Third Affiliated Hospital of Wenzhou Medical College and the People’s Hospital of Deyang City. Each of the participants provided informed consent (in the Chinese language) before participating in this study. In most circumstances, the consent could be obtained directly from the patients. For patients who cannot write or read Mandarin, the nurse will record the patient’s verbal consent, including the experimental information provided to the participants and the time of verbal consent, another nurse is responsible for monitoring and signing the informed consent. The oral or written informed consent will be kept in the participant’s hospital chart.

### Study population

We consecutively enrolled 236 acute IS patients with significant (≥ 50%) symptomatic carotid stenosis and 378 acute IS patients without carotid stenosis from August 2012 to June 2016. These participants were all suffered from first-ever IS and admitted to the above two hospitals within 3 days of the index stroke onset. The diagnostic criteria of acute IS in this study were: (1) sudden onset and focal neurological deficit of vascular origin; (2) symptoms/signs last for at least 24 h; (3) have relevant cerebral lesions on diffusion weighted magnetic resonance imaging (DWI); (4) Brain computed tomography scan/magnetic resonance imaging excludes cerebral hemorrhage. Common electrocardiogram (ECG), 24-h Holter ECG and echocardiogram were measured in all patients. The inclusion criteria for patients were: (1) age ≥ 40 years old; (2) Infarct lesions were located in internal carotid artery system territory (anterior circulation infarction). Exclusion criteria were: (1) atrial fibrillation or cardiac embolism; (2) IS caused due to unknown factors; (3) Infarct lesions were located in vertebrobasilar system territory (posterior circulation infarction); (4) treatment by carotid stent or carotid endarterectomy; (5) patients with 15–49% carotid stenosis or intracranial arterial stenosis or asymptomatic carotid stenosis or vertebrobasilar artery stenosis; (6) Refuse to participate for personal factors; (7) Any relatedness between these patients.

### Carotid ultrasonography

Color duplex imaging (Acuson Sequoia Apparatus type 512, 7.5-MHz probe, Berlin, Germany) was used to evaluate the extent of carotid stenosis. According to the conventional scanning and reading protocols [8, 20], the morphological lumen reduction assessment in diameter was combined with peak systolic velocities at the location of the stenosis and the internal carotid artery/common carotid artery (ICA/CCA)ratio. For example, patients with peak systolic velocities of 120-220 cm/s and ICA/CCA ratio of 1.5–3.7 were defined as 50 to 69% stenosis; and peak systolic velocities of > 220 cm/s as well as ICA/CCA ratio of > 3.7 were defined as 70 to 99% stenosis [20]. The investigators were blinded to the patient’s clinical data and independently graded for carotid stenosis. We assessed the reproducibility of the extent of carotid stenosis in 28 patients. The 28 patients were randomly selected from enrolled patients with carotid stenosis through the randomization office in our hospital by the Internet. The coefficients of intra-observer and inter-observer variations for the degree of carotid stenosis were 7.4 and 7.8%, respectively. The detailed procedures for evaluating carotid stenosis, coefficients of intraobserver variation and interobserver variation were described in our previous study [8]. Furthermore, 80 patients with duplex-based stenosis were also evaluated blindly by conventional angiography [21]. Exact agreement was found in 85% of these patients, and there were no major (> 1 stenosis group up or down) disagreements.

Symptomatic carotid stenosis (≥ 50% stenosis) were defined as they experienced an ipsilateral IS in carotid territory. Patients without carotid stenosis were selected from IS patients at same period (between August 2012 and June 2016). Patients without carotid stenosis were defined as less than 15% carotid stenosis [22]. Our current study mainly investigate the association between variants in platelet activation-relevant genes and symptomatic carotid stenosis in IS patients. Also we referred the study of Worrall et al [22]. For excluding the effect of 15–49% carotid stenosis or intracranial arterial stenosis on the results, thus, the patients with 15–49% carotid stenosis or intracranial arterial stenosis were also excluded in this study.

### Evaluation of risk factors

Take record of atherosclerotic risk factors for all participants, including age, gender, weight, smoking, hypertension, diabetes, and myocardial infarction [MI]. Other indicators were detected by a blood sample which includes blood glucose, hemoglobin A1c, homocysteine, total serum cholesterol (TC), triglycerides (TG) and low-density lipoprotein (LDL-C). Hyperlipidemia was defined as TC > 200 mg/dL, TG > 180 mg/dL or use of lipid-lowering medication. Hypertension was defined as a mean of three independent measures of blood pressure of ≥140/ 90 mmHg or the use of antihypertensive drugs. Diabetes mellitus was defined by a fasting glucose level of > 7.8 mmol/L or that of > 11.1 mmol/L 2 h after an oral glucose challenge. Alcohol intake was defined as drinking alcohol at least 12 times in the past year and Cigarette smoking was defined as having smoked at least one cigarette a day for more than 1 year. MI was diagnosed as the presence of at least two of these criteria: prolonged angina > 30 min; electrocardiographic evidence of infarction; total creatinine kinase isoenzyme elevation more than twice the upper limit of normal.

### Genotyping and selection of SNPs

The 11 SNPs of platelet activation-relevant genes were selected from NCBI database (http://www.ncbi.nlm.nih.gov/SNP), according to the following standard: (1) each SNP had been evaluated in previous studies; (2) the SNPs with minor allele frequency > 0.05; (3) the SNPs lead to changes of amino acid. Venous blood (3 mL) was drawn from an arm vein into a sterile tube containing ethylenediaminetetraacetic acid. Genomic DNA was extracted from peripheral blood using a modified phenol/chloroform method and purified using the UNIQ-10 kit (Sangon Biotech Co., Ltd. Shanghai, China) [7, 8]. As the previous study described, adopting matrix-assisted laser desorption ionization through time-of-flight mass spectrometry to detect genotypes of the 11 variants [7, 8, 15, 16].

### Measurement of platelet activation

Venous blood (6 mL) was drawn from the anterior elbow vein at admission. Light transmittance aggregometry was used to measure platelet aggregation which induced by arachidonic acid (AA) or adenosine diphosphate (ADP) (Helena Laboratories, Beaumont, TX, USA). Platelet-leukocyte aggregates were measured using FC 500 MPC flow cytometry (Beckman Coulter Ltd) and direct fluorescent markers (Coulter Immunotech, Krefeld, Germany). The detailed procedures for measuring platelet-leukocyte aggregates and platelet aggregation were described in our previous studies [15, 16].

### Statistical analysis

According to a suggested sample size requirement for gene-gene interactions [23], we calculated that our sample size of 230 patients with symptomatic carotid stenosis and 370 patients without carotid stenosis would provide 80% power at the 5% significance level calculated using three genetic models: the additive model, the dominant model, and the recessive model.

Statistical analysis was performed using SPSS 16.0 (SPSS Inc., Chicago, IL, USA). The deviation of the Hardy-Weinberg equilibrium for genotype frequencies was analyzed by χ^2^ test. Comparison of continuous variables was using Student’s t-test, meanwhile, discrete variables were assessed using χ^2^ test between patients with and without carotid stenosis. Differences of genotype frequencies between patients with and without carotid stenosis were also compared using χ^2^-test. Gene-gene interaction was analyzed using statistical software GMDR Beta, version 0.7 (www.healthsystem.virginia.edu/internet/addiction-genomics/Software) [19], as described in our previous studies [15, 16].

Subsequently, multivariate logistic regression was performed to adjust covariate variables, such as age, gender, hypertension, diabetes, smoking, hyperlipidemia, homocysteine, and hemoglobin A1c, and to assess the independent contribution of the 11 variants and interactions among these variants on carotid stenosis, and odds ratio (OR) with 95% confidence intervals (CI) were calculated. All tests were two sided, and the threshold level of *P* < 0.05 denoted statistical significance.

## Results

### Baseline characteristics in patients with and without carotid stenosis

There were no significance differences in conventional risk factors and previous drugs treatments (including statins, antiplatelet therapy, antihypertensive drugs hypoglycemic drugs) between patients with and without carotid stenosis (Table 1). However, compared with patients without carotid stenosis, the platelet aggregation induced by AA or ADP and platelet-leukocyte aggregates were significantly higher in patients with carotid stenosis (Table 1).

**Table 1:** Characteristics of ischemic stroke patients with or without carotid stenosis

Characteristics	Carotid stenosis (*n* = 236)	Non-carotid stenosis (*n* = 378)	*P* value
Age (years)	69.1 ± 12.8	68.8 ± 15.9	0.607
Men (n, %)	142 (60.2)	222 (58.7)	0.597
Hypertension (n, %)	192 (81.4)	301 (79.6)	0.796
Diabetes mellitus (n, %)	81 (34.3)	124 (32.8)	0.379
Previous MI (n, %)	4 (1.7)	6 (1.6)	0.996
Cigarette smoking (n, %)	94 (39.8)	148 (39.1)	0.962
Alcohol intake (n, %)	108 (45.8)	170 (45.0)	0.896
Body mass index (kg/m2)	24.2 ± 3.7	24.0 ± 4.2	0.943
Hyperlipidemia(n, %)	129 (54.7)	199 (52.6)	0.467
Fasting bloodglucose (mM)	6.8 ± 3.2	6.6 ± 4.3	0.411
Hemoglobin A1c (%)	6.4 ± 2.8	6.2 ± 2.7	0.357
Homocysteine (mM)	15.1 ± 5.5	14.7 ± 5.8	0.108
Previous drugs treatments (n, %)			
Antihypertensive drugs	131 (55.5)	208 (55.0)	0.996
Hypoglycemic drugs	60 (25.4)	100 (26.5)	0.994
Statins	50 (21.2)	81 (21.4)	0.999
Antiplatelet drugs	71 (30.1)	115 (30.4)	0.998
Platelet aggregation (%)			
AA-induced	90.3 ± 13.2	86.2 ± 14.7	< 0.001
ADP-induced	89.7 ± 13.4	86.3 ± 13.6	0.002
Platelet-leukocyte aggregates (%)			
Leukocyte	26.8 ± 6.5	24.2 ± 6.6	< 0.001
Neutrophil	27.1 ± 6.6	24.3 ± 7.2	< 0.001
Monocyte	26.7 ± 5.9	23.7 ± 6.5	< 0.001
Lymphocyte	26.3 ± 6.3	24.4 ± 6.4	< 0.001

*MI* myocardial infarction, *AA* arachidonic acid, *ADP* adenosine diphosphate

### Genotype distributions in patients with and without carotid stenosis

The genotype distributions of the 11 variants were consistent with the Hardy-Weinberg Equilibrium (all *P* > 0.05). The frequency of *TXA2R* rs1131882TT, *P2Y1* rs1371097TT, *TXAS1* rs41708TT, and *GPIIIa* rs2317676GG was significantly higher in patients with carotid stenosis than those without carotid stenosis (Table 2). However, after adjusting for potential confounding variables, the rs1131882TT, rs1371097TT, rs41708TT, and rs2317676GG did not independently affect the risk for carotid stenosis.

**Table 2:** Genotype distribution of patients with or without carotid stenosis (*n,* %)

	carotid stenosis (n = 236)	Non-carotid stenosis (n = 378)	*P* value	OR^a^ (95% CI)
*TXA2R* (rs1131882)				
CC	62 (26.3)	132 (34.9)	0.024	1.21 (0.95–1.98)
CT	105 (44.5)	175 (46.3)		
TT	69 (29.3)	71 (18.8)		
*TXAS1* (rs2267679)				
CC	7 (3.0)	12 (3.2)	0.786	0.94 (0.71–1.63)
CT	39 (16.5)	68 (18.0)		
TT	190 (80.5)	298 (78.8)		
*TXAS1* (rs194149)				
AA	39 (16.5)	63 (16.7)	0.673	0.83 (0.663–1.72)
AG	118 (50.0)	181 (47.9)		
GG	79 (33.5)	134 (35.4)		
*TXAS1* (rs41708)				
GG	116 (49.2)	228 (60.3)	0.011	1.23 (0.58–2.34)
GT	66 (27.9)	113 (29.9)		
TT	54 (22.9)	37 (9.8)		
*P2Y1*(rs701265)				
AA	127 (53.8)	202 (53.4)	0.989	0.67 (0.82–1.24)
AG	70 (29.7)	113 (29.9)		
GG	39 (16.5)	63 (16.7)		
*P2Y1*(rs1439010)				
AA	127 (53.8)	202 (53.4)	0.886	0.93 (0.83–1.68)
AG	71 (30.1)	113 (29.9)		
GG	38 (16.1)	63 (16.7)		
*P2Y1*(rs1371097)				
CC	141 (59.7)	203 (53.7)	0.006	1.07 (0.96–2.18)
CT	38 (16.1)	120 (31.7)		
TT	57 (24.2)	55 (14.6)		
*P2Y12*(rs16863323)				
CC	59 (25.0)	102 (27.0)	0.155	0.95 (0.92–1.66)
CT	66 (28.0)	132 (34.9)		
TT	111 (47.0)	144 (38.1)		
*P2Y12*(rs9859538)				
GG	160 (67.8)	256 (67.7)	0.966	0.88 (0.67–1.45)
AG	50 (21.2)	84 (22.2)		
AA	26 (11.0)	38 (10.1)		
*GPIIIa* (rs2317676)				
AA	135 (57.2)	234 (61.9)	0.012	1.04 (0.76–2.14)
AG	47 (19.9)	106 (28.2)		
GG	54 (22.9)	38 (10.1)		
*GPIIIa* (rs11871251)				
AA	89 (37.7)	144 (38.1)	0.962	0.82 (0.53–1.37)
AG	78 (33.1)	129 (34.1)		
GG	69 (29.2)	105 (27.8)		

*OR* odds ratio, *CI* confidence interval

^a^OR adjusted for age, gender, hypertension, diabetes, smoking, hyperlipidemia, homocysteine and hemoglobin A1c

### Gene-gene interactions and carotid stenosis

High-order gene-gene interactions were performed by the GMDR method. After adjustment with confounding variables, the best model for carotid stenosis was rs1131882, rs1371097 and rs2317676, which scored 9/10 for the sign test and 10/10 for cross-validation consistency (*P* = 0.022, Table 3). For computing of each variant, the one-locus model was applied in turn, and the empirical *P* values of prediction errors by permutation testing were 0.028.

**Table 3:** Comparison of the best models, prediction accuracies, cross-validation consistencies, and *P* values for carotid stenosis identified by GMDR

Best model^a^	Training balanced accuracy	Testing balanced accuracy	Cross-validation consistency	Sign test (*P*) †
1	0.476	0.531	7/10	7 (0.427)
1, 2	0.614	0.667	9/10	9 (0.156)
1, 2, 3	0.672	0.686	10/10	9 (0.022) ‡
1, 2, 3, 4	0.497	0.598	8/10	8 (0.421)
1, 2, 3, 4, 5	0.623	0.546	7/10	7 (0.712)
1, 2, 3, 4, 5, 6	0.587	0.623	8/10	7 (0.643)
1, 2, 3, 4, 5, 6, 7	0.511	0.472	9/10	5 (0.745)
1, 2, 3, 4, 5, 6, 7, 8	0.632	0.643	5/10	7 (0.524)
1, 2, 3, 4, 5, 6, 7, 8, 9	0.586	0.597	7/10	8 (0.628)
2, 3, 4, 5, 6, 7, 8, 9, 10	0.617	0.486	8/10	6 (0.586)
1, 2, 3, 4, 5, 6, 7, 8, 9, 10,11	0.523	0.677	6/10	7 (0.467)

^a^rs1131882, rs2317676, rs1371097, rs194149, rs2267679, rs41708, rs701265, rs1439010, rs16863323, rs9859538, rs11871251 are symbolized as 1–11, respectively

GMDR, generalized multifactor dimensionality reduction

† *P* adjusted for age, gender, hypertension, diabetes, smoking, hyperlipidemia, homocysteine and hemoglobin A1c

‡best model for combination of different variant was defined as *P <* 0.05 after adjustment with confounding variables

### Association of different genotype combinations of rs1131882, rs1371097 and rs2317676 with carotid stenosis risk

Subsequently, we assessed the relationship between different genotype combinations of the three variants and the risk of carotid stenosis. Compared to patients carrying wild type genotype (ie. rs1131882CC, rs1371097CC and rs2317676AA), three interactions making more contribution to carotid stenosis risk were among rs1131882TT, rs1371097TT and rs2317676GG; rs1131882TT, rs1371097CT/TT and rs2317676GG; and rs1131882TT, rs1371097CT and rs2317676AG (Table 4). The above three combination genotypes of rs1131882, rs1371097 and rs2317676 were considered as high-risk interactive genotypes. The other combination genotypes of rs1131882, rs1371097 and rs2317676 did not reach statistical significance (*P* > 0.05), and were defined as low-risk interactive genotypes (Table 4).

**Table 4:** Associations between genotype combinations and carotid stenosis

Rs1131882	CC	TT	TT	TT	CT	TT	TT, CT	TT, CT
Rs1371097	CC	TT	CT, TT	CT	CT	TT	TT	TT, CT
Rs2317676	AA	GG	GG	AG	AG	AG, GG	GG	GG, AG
OR	1 ^a^	2.76	1.99	2.11	1.06	1.14	1.17	1.05
95% CI	–	1.27–7.89	1.03–5.12	1.08–6.15	0.87–1.77	0.88–2.23	0.94–2.87	0.81–1.92
*P* value	–	0.002†	0.023†	0.018†	0.213‡	0.385‡	0.165‡	0.467‡

^a^The wild type genotype for each genetic factor was used as the reference OR. OR, odds ratio; CI, confidence intervals

† high-risk interactive genotypes were defined as there were significant associations between different combination of genotypes and carotid stenosis after adjustment with confounding variables (*P <* 0.05)

‡ low-risk interactive genotypes were defined as there were no significant associations between different combination of genotypes and carotid stenosis after adjustment with confounding variables (*P >* 0.05)

### Associations of genetic variants with platelet activation

There were no significant differences in platelet-leukocyte aggregates and platelet aggregation among genotypes of the 11 variants. While compared with patients carrying low-risk interactive genotypes of rs1131882, rs1371097 and rs2317676, the platelet-leukocyte aggregates and platelet aggregation were significantly higher in patients carrying high-risk interactive genotypes (Table 5).

**Table 5:** Association of high-risk interactive genotypes with platelet aggregation and platelet-leukocyte aggregates

1	Platelet aggregation (%)		Platelet-leukocyte aggregates (%)			
	AA-induced	ADP-induced	Leukocyte	Neutrophil	Monocyte	Lymphocyte
High-risk interactive genotypes †						
Yes (*n* = 180)	89.4 ± 11.5	89.2 ± 12.3	24.9 ± 5.7	25.8 ± 5.8	27.2 ± 6.7	26.5 ± 5.4
No (*n* = 434)	84.3 ± 14.6	84.7 ± 13.7	22.3 ± 6.4	23.5 ± 6.2	24.1 ± 7.3	24.8 ± 6.6
*P* value	< 0.001	< 0.001	< 0.001	< 0.001	< 0.001	< 0.001

*AA* arachidonic acid, *ADP* adenosine diphosphate

† high-risk interactive genotypes were defined as there were significant associations between different combination of genotypes and carotid stenosis after adjustment with confounding variables (*P <* 0.05)

### Risk factors of carotid stenosis

Multivariate logistic regression analysis was performed to assess risk of carotid stenosis. The high-risk interactions were assigned as one, and low-risk interactions were assigned as zero. The results revealed that the high-risk interactions among rs1131882, rs1371097 and rs2317676 were independently associated with higher risk for carotid stenosis, after adjusting for hypertension, diabetes mellitus, hyperlipidemia, homocysteine and hemoglobin A1c (OR, 2.69, 95% CI: 1.22–7.33, *P* = 0.003, Table 6).

**Table 6:** Multivariate analysis of the major risk factors for carotid stenosis

Risk factor	OR^a^	95% CI	*P* value
Hypertension	1.91	1.03–4.02	0.031
Diabetes mellitus	1.77	1.02–2.92	0.039
Homocysteine	0.92	0.85–1.54	0.355
Hyperlipidemia	0.88	0.79–1.31	0.734
Hemoglobin A1c	0.93	0.85–1.37	0.546
Previous statins	0.79	0.54–1.12	0.473
Previous antiplatelet therapy	0.83	0.72–1.26	0.502
High-risk interactions	2.69	1.22–7.33	0.003

*OR* odds ratios, *CI* confidence interval

^a^OR for Homocysteine, Hemoglobin A1c, Platelet aggregation and Platelet-leukocyte aggregates means per 1-Standard Deviation increase

## Discussion

In this study, we found that the high-risk interactions among *TXA2R* rs1131882, *P2Y1* rs1371097 and *GPIIIa* rs2317676 were associated with higher platelet activation, and independently associated with higher risk of carotid stenosis in IS patients.

Atherosclerosis is a multifactorial disease. Platelet activation may play an essential role in the pathophysiology of atherosclerosis and arterial thrombosis [9, 10, 24, 25]. Our previous studies have shown that polymorphisms in genes involved in platelet activation could increase the risk of IS [16, 17], and affect responsiveness of antiplatelet drugs [18]. Although the 11 variants were not independently associated with carotid stenosis, the GMDR analysis revealed that the high-risk interactions among rs1131882, rs1371097 and rs2317676 were independently associated with higher risk of carotid stenosis in this study. This indicated that single-locus analytical approach seemed unsuitable for the complex genetic etiology of carotid stenosis. Atherosclerosis may be caused by gene-gene or gene-environment interactions [26, 27]. Therefore, genetic risk of carotid stenosis could be enhanced by investigating gene-gene interactions via alternative analytical methods, e.g. GMDR approach [19].

The GMDR analysis offered a noteworthy finding in this study that gene-gene interactions among platelet activation-relevant genes played an interesting synergistic role which contributed to carotid stenosis risk. The risk of carotid stenosis was increased by 2.69 times in patients with high-risk interactive genotypes of rs1131882, rs1371097 and rs2317676 than those with low-risk interactive genotypes, indicating the interactions of specific platelet activation-relevant gene conferred a higher risk of carotid stenosis.

The pathophysiological mechanisms of the three variants interactions are unclear. Our current results showed that platelet activation (platelet-leukocyte aggregates and platelet aggregation) was significantly higher in patients carrying high-risk interactive genotypes compared with those carrying low-risk interactive genotypes. Thus, one possible explanation is that the rs1131882, rs1371097 and rs2317676 encode TXA2R, P2Y1 and GPIIIa, respectively, which participate in the process of platelet activation. Platelet activation plays an important role in the pathophysiological mechanisms of atherosclerosis [9, 10]. TXA2 is a platelet activator and vasoconstrictor. TXA2, TXA2R, platelet membranes receptors, and glycoprotein IIIa are all very crucial for platelet activation [11–13]. Our previous studies and some other studies have shown that polymorphisms of *TXA2R* and *TXAS1* genes are associated with platelet activation, and independently associated with the risk of carotid plaque vulnerability, carotid or intracranial arterial stenosis and IS [7, 15, 28–30]. In healthy subjects, platelet activation was associated with a haplotype of the *P2Y12* gene [31]. The SNPs in *P2Y12, P2Y1* and *GPIIIa* genes were also associated with high platelet activation and attenuated response to antiplatelet drug [32–34]. Although, single locus analysis did not demonstrate the associations of variants in C*OX-2* rs20417, *P2Y1* rs1371097 and *GPIIIa* rs2317676 with platelet aggregation and aspirin resistance, the interaction of the three variants contributed to aspirin resistance in IS patients, and platelet aggregation was significantly higher in patients carrying high-risk interactive genotypes than those with low-risk interactive genotypes [35]. Clopidogrel is a clinically important oral antiplatelet agent for the treatment of IS. Our previous study revealed that there was a significant gene-gene interaction among CYP2C19*2 (rs4244285), *P2Y12* (rs16863323), and *GPIIIa* (rs2317676), which contributed to clopidogrel resistance in IS patients receiving clopidogrel therapy [36]. Thus, according to our current results and some other studies, we reason that the high-risk interactions among *TXA2R* rs1131882, *P2Y1* rs1371097 and *GPIIIa* rs2317676 could provide these individuals with higher platelet activation than those without this particular high-risk interactions, thereby increasing the risk for carotid stenosis.

In spite of these novel findings in this study, several limitations should be noted as well. Firstly, carotid stenosis was only assessed by ultrasound in this study. Although ultrasonography is highly sensitive for detecting carotid stenosis. Computed tomographic angiography (CTA) and high-resolution magnetic resonance imaging (HR-MRI) could provide more information for carotid stenosis [21, 37, 38]. Thus, it is necessary to assess carotid stenosis using CTA or HR-MRI and validate our current findings in future. Second, due to the limited sample size and two-center study, our results may not represent the full spectrum of Chinese populations. Our findings must be validated in larger, multi-center studies. Third, the morphological characters of carotid plaque such as echo-free plaque, ulcerative plaque are very important risk factors for IS [39]. The main aim of this study was to investigate the associations between variants in platelet activation-relevant genes and carotid stenosis. Thus, we did not assess the relations between these variants and carotid plaque vulnerability in this study, which should be assessed in the future study. Finally, although we genotyped known functional variants of platelet activation-relevant genes, some rare functional variants were not investigated in this population. Furthermore, lack of an independent sample for replication was also a limitation in this study. Thus, a study with a larger sample of genetic variants should be done and help to elucidate the full extent of gene-gene interactions effects on carotid stenosis.

## Conclusion

In present study, we found that there is no independent association between 11 variants of platelet-activating genes and the risk of carotid stenosis using single-locus analysis. However, the GMDR analysis showed that there was a gene-gene interaction among *TXA2R* rs1131882, *P2Y1* rs1371097 and *GPIIIa* rs2317676 which significantly increased the risk of carotid stenosis. The high-risk interactions among the three variants were associated with platelet activation, and independently associated with the higher risk for carotid stenosis.

## Abbreviations

AA: arachidonic acid
ADP: adenosine diphosphate
CI: confidence intervals
CTA: computed tomographic angiography
GMDR: generalized multifactor dimensionality reduction
GPIIIa: glycoprotein IIIa
HR-MRI: high-resolution magnetic resonance imaging
IS: ischemic stroke
LDL-C: low-density lipoprotein cholesterol
MI: myocardial infarction
OR: odds ratio
SNPs: single nucleotide polymorphisms
TC: total cholesterol
TG: triglycerides
TXA2: thromboxane A2
TXA2R: TXA2 receptor
TXAS: thromboxane synthase
